# Case Report: Langerhans cell histiocytosis involving the cervical spine in an adult patient

**DOI:** 10.12688/f1000research.139360.1

**Published:** 2023-09-21

**Authors:** Dipak Chaulagain, Volodymyr Smolanka, Andriy Smolanka, Taras Havryliv

**Affiliations:** 1 Neurosurgery, Uzhhorod National University, Uzhhorod, Ukraine

**Keywords:** Langerhans cell histiocytosis, Histiocytosis, Cervical spine

## Abstract

Langerhans Cell Histiocytosis (LCH) is a typically benign disorder that affects infants predominately, with adult occurrence being uncommon. We discuss the case of a 22-year-old guy who visited our clinic complaining of three months of acute nape pain and upper limb radiculopathy. Notably, the patient had no history of trauma, fall injuries, or tuberculosis. Radiological tests identified a single osteolytic lesion within the C3 vertebral body. The lesion was removed, an anterior C3 corpectomy and discectomy were performed, and the patient’s spine was reconstructed with a titanium cage and plating. The patient’s nape discomfort and radiculopathy vanished almost instantly after surgery. A definitive diagnosis of LCH was confirmed through histological examination. This case report illustrates the unusual and uncommon occurrence of LCH at the C3 vertebral body, for which fusion surgery was the only viable therapeutic option. The patient’s recovery from radiating pain following the surgical procedure demonstrates the effectiveness of the intervention. LCH in the cervical spine is rather rare, but it is nevertheless important to be aware of the possibility of developing it.

## Introduction

Langerhans cell histiocytosis (LCH) is a rare disease that causes Langerhans cells to multiply in a way that isn’t normal. This causes sores on the skin, the bones, the liver, the spleen, and the lymph nodes. ^1^^,^^2^ The localized form of LCH is the most common presentation, typically manifesting as a solitary bone lesion predominantly seen in children. ^3^^,^^4^ In about 7–15% of cases, the spine is affected. The thoracic spine is most often affected (54%), followed by the lumbar spine (35%), and then the cervical spine (11%). ^5^

The clinical presentation of LCH can vary widely, ranging from self-healing bone lesions to life-threatening multi-system involvement. Consequently, determining the most appropriate treatment approach poses a significant challenge, as therapeutic options span from watchful waiting to aggressive interventions. ^6^^,^^7^

Lesions of the adult spine in the context of Langerhans cell histiocytosis do not appear to have any particular therapeutic recommendations or treatment regimens in the existing research. In addition, there is a paucity of research devoted to surgical treatments for LCH of the spine, therefore there is little in the way of specifics or established protocols for how to treat this condition in adults.

In this report, we describe a case of adult Langerhans cell histiocytosis (LCH) involving the C3 vertebrae, a location seldom affected by this disease. The patient initially presented to our hospital with pain in the nape region, which prompted the decision to pursue surgical intervention. During the surgery, an anterior lesion was removed, an anterior corpectomy with discectomy was done, and then the area was put back together with a titanium cage and plating.

## Case report

The Regional Clinical Centre of Neurosurgery and Neurology in Uzhhorod, Ukraine, had a visit from a 22-year-old male patient of Ukrainian nationality. The patient, who is now pursuing studies, reported experiencing neck discomfort for a duration of three months. There was no familial history indicating the presence of similar symptoms. The patient’s neurological problems did not improve in spite of getting non-steroidal anti-inflammatory medicines and physical therapy. The cranial nerves were confirmed to be intact, and no localized weakening was observed during the motor assessment. The patient exhibited normal physiological reflexes. However, he experienced increasing discomfort in the posterior neck region.

**Figure 1.**
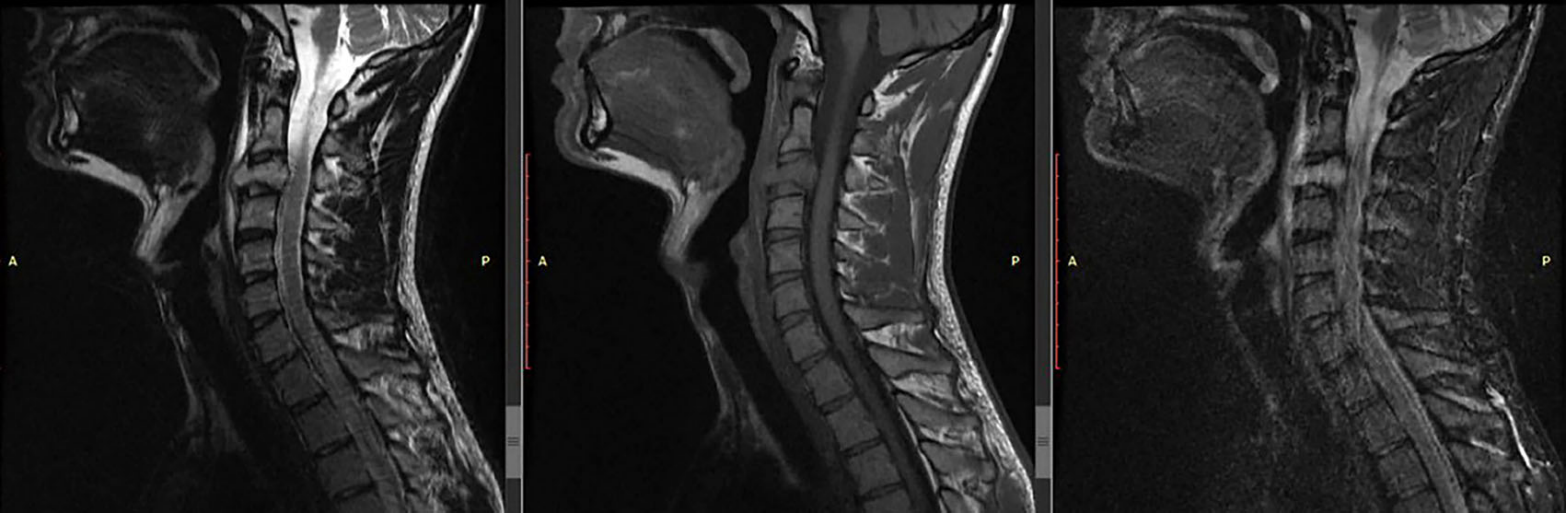
Pre-operative MRI showed C3 vertebral body collapse with epidural and prevertebral soft tissue collection causing cord.

A magnetic resonance imaging (MRI) of the cervical spine revealed that there were anomalies in the C3 vertebral body. An enlarged mass-like lesion may be seen on contrast-enhanced T1-weighted imaging; this lesion has migrated to the anterior epidural region, which reveals central spinal stenosis ( Figure 1).

**Figure 2.**
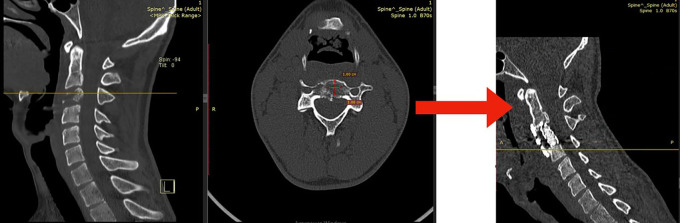
A computed tomography (CT) image taken after surgery reveals a titanium cage and plating were used in conjunction with an anterior cervical corpectomy and discectomy.

The patient had a titanium cage and plating restoration after a discectomy and anterior corpectomy at C3 ( Figure 2).

These specimens revealed fragments of connective tissue and bone with a histiocytic lesion upon microscopic examination. The histomorphological aspect and the immunohistochemical staining profile argue for a Langerhans cell histiocytosis. LCH was the histological diagnosis. Positive immunohistochemical stains were observed for CD1a, S-100, CD68, and KiM1p.

**Figure 3.**
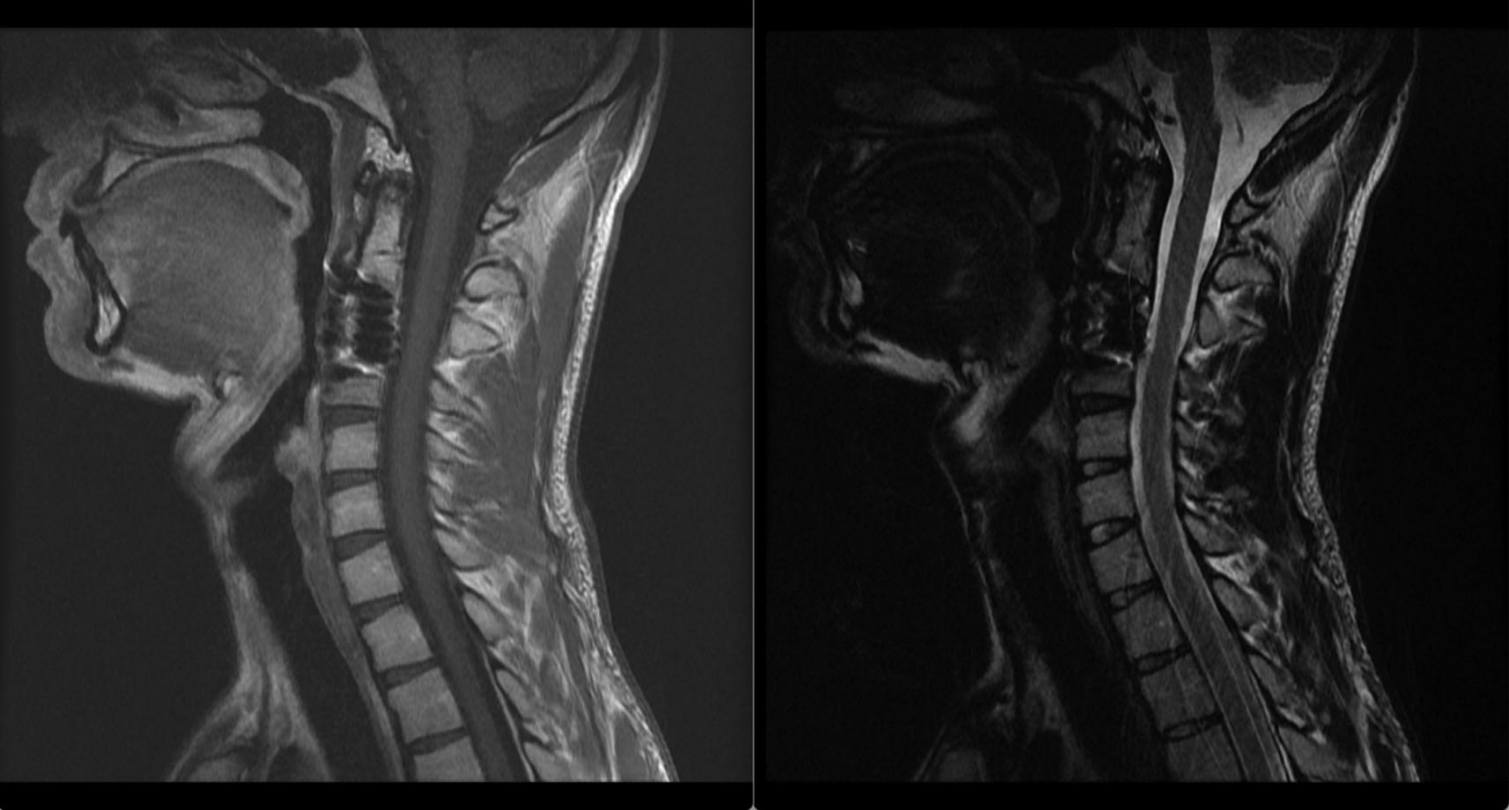
One month after surgery, follow-up MRI scans showed no evidence of tumor growth in the C3 vertebral body.

Following the surgical procedure, there was a full recovery from the pain in the neck. One month following the operation, MRI scans revealed that the C3 vertebral body had not shown any signs of new tumor development ( Figure 3). We found no evidence of a previous history of problems or a worsening of the symptoms.

## Discussion

Interleukin-17A, among other inflammatory cytokines, is recognized to have a role in the development of Langerhans cell histiocytosis (LCH). ^8^^,^^9^ Estimates place the incidence of LCH in adults between 1 and 2 cases per million population, while in children, the incidence ranges from 2 to 10 cases per million. However, it is likely that these numbers underestimate the true incidence of the disease. ^10^ In the case presented here, the patient’s age of 22 years suggests the possibility of a delayed diagnosis, which may have contributed to the later presentation of the disease.

Langerhans cell histiocytosis (LCH) exhibits a predilection for various skeletal sites, with the skull being the most commonly affected (26%), followed by the ribs (12%), mandible and upper extremities (9%), and other bones of the extremities (11%). ^11^ In terms of spinal involvement, LCH is relatively infrequent, accounting for only a small percentage of cases. Only 6.5% (14 cases) of the 214 cases reported by Bunch et al. were localized in the spine. Spinal lesions mostly affect the vertebral bodies, with the thoracic spine (54% prevalence) being greater than the lumbar (35% prevalence) and cervical (11% prevalence). ^11^ LCH is so uncommon that determining its prevalence as a single cervical lesion is challenging. Just 0.02% of 342 cases evaluated by Howarth et al. ^9^ had spinal involvement, and of those, exclusively in the cervical vertebrae. The fact that LCH rarely presents as a single lesion in the cervical spine is highlighted by this.

This patient is a rare example of an adult with Langerhans cell histiocytosis (LCH) of the cervical spine. Radiological alterations shown on imaging investigations, along with certain pathological and immunohistochemical results, are often used to make a diagnosis of LCH. Confirming the presence of LCH and differentiating it from other illnesses with comparable clinical presentations rely heavily on these methods of diagnosis.

Histopathological examination of Langerhans cell histiocytosis (LCH) under the microscope reveals eosinophils, large cells, neutrophils, foamy cells, and fibrosis, among other possible findings. As compared to other cell types, Langerhans cells may be identified thanks to their positive immunostaining both CD1a with S-100 protein. ^12^ In addition to aiding in a diagnosis of LCH, these microscopic features and immunohistochemistry markers can be used to set LCH apart from other histiocytic illnesses and inflammatory syndromes.

Determining an optimal treatment strategy for Langerhans cell histiocytosis (LCH) remains a highly controversial issue. Previous literature suggests that the prognosis for vertebral involvement is generally considered benign, with favorable outcomes. Specifically, there have been no reported cases of significant neurological deficits resulting from vertebral spine involvement in LCH. ^13^ These findings imply that the vertebral types of LCH typically exhibit a less aggressive disease course and are associated with a relatively good prognosis.

In a study by Monalti et al., two patients with a solitary eosinophilic granuloma (EG) of the spine experienced rapid pain alleviation after receiving corticosteroid infiltrations. However, post-interventional imaging did not match the clinical manifestations observed in these patients. In particular, computed tomography (CT) imaging revealed a reduction in lesion size in one patient while the morphology of the vertebral lesion remained unchanged in the second patient. ^14^ There are a number of methods for dealing with Langerhans cell histiocytosis (LCH), including close surveillance, NSAIDs, chemotherapy, radiation, intralesional steroid injection, surgical removal of the lesion, and spinal repair. ^15^

Langerhans cell histiocytosis (LCH) indications for radical surgery remain inadequately documented. Nevertheless, given the patient’s presentation with radiculopathy, we decided to pursue radical surgical intervention. This decision was made based on the clinical condition of the patient and the urgency need to relieve compression and preserve neurological integrity. Prior studies have reported successful resolution of Langerhans cell histiocytosis (LCH) via observation, surgery, chemotherapy, and radiation therapy, among others. Individual patient characteristics, the severity of the disease, and the presence of associated symptoms or complications determine which of these treatment options are employed. Thus, a titanium cage and plating reconstruction with anterior cervical C3 corpectomy and discectomy was performed. Further research is needed with a larger sample size to provide firm guidelines and criteria for LCH surgical treatment in the adult cervical spine. The sample tissue was examined under a microscope to confirm the diagnosis of LCH.

Following the surgical intervention, the patient experienced complete resolution of posterior neck pain. Subsequent postoperative MRI studies conducted one month after the surgery indicated no evidence of tumor re-growth and the absence of any lesions or signs of tumor regrowth within the fusion construct. These results suggest that surgical decompression may be a viable alternative for relieving pain and preventing neurologic impairment when other treatments have failed.

## Conclusion

We suggest an anterior cervical corpectomy with discectomy and titanium cage and plating for the treatment of adult Langerhans cell histiocytosis (LCH). This technique, which is performed through a direct, anterior access to the cervical vertebrae, has the potential to alleviate pain, achieve stability, and restore function, particularly in cases involving significant cortical bone destruction and concomitant neurological deficits in the cervical spine. It is essential to note, however, that these results are founded on a singular case study; therefore, additional research employing more extensive research methodologies is necessary. Further research is required to ascertain whether LCH in the adult cervical spine should be surgically treated and how the procedure should be carried out.

## Consent

We are pleased to confirm that we have received written consent from the patient, for the publication of their clinical details and images in the manuscript. This consent statement is a vital aspect of our submission, and we are committed to upholding the highest ethical standards in medical research. The consent statement, duly signed by the patient’s parent, has been provided. This statement outlines the patient and parent of patient’s clear understanding and agreement regarding the publication of their clinical information and images for educational and research purposes. We would like to reiterate our commitment to maintaining patient confidentiality and ensuring that all ethical guidelines are adhered to throughout the publication process.

## Data Availability

No data are associated with this article.
