# Supplementary material for: Case Report: Langerhans cell histiocytosis involving the cervical spine in an adult patient
Source: F1000Res. 2023 Sep 21;12:1185. [Version 1] doi: 10.12688/f1000research.139360.1 (PMC11162520; doi:10.12688/f1000research.139360.1)
Supplement: Peer review history

# Reviewer report: Reviewer response for version 1

**DOI:** 10.5256/f1000research.152631.r246281

**Published:** 2024-06-08

**Authors:** Mohammad Alfawareh

**Affiliations:** 1 King Hussain Cancer Center, Amman, Jordan

well described well formatted manuscript

 the introduction gave details about the disease and how common histiocytosis  

 case report well described the case scenario, covering all the needed details

 images gave great explanation to the case

 post operative CT scan well informative 

 discussion well constructed and exploring possible deferential and management details were included

Are enough details provided of any physical examination and diagnostic tests, treatment given and outcomes?

Yes

Is the case presented with sufficient detail to be useful for other practitioners?

Yes

Is sufficient discussion included of the importance of the findings and their relevance to future understanding of disease processes, diagnosis or treatment?

Yes

Is the background of the case’s history and progression described in sufficient detail?

Yes

Reviewer Expertise:

Spine Deformiy and Tumors

I confirm that I have read this submission and believe that I have an appropriate level of expertise to confirm that it is of an acceptable scientific standard.

# Reviewer report: Reviewer response for version 1

**DOI:** 10.5256/f1000research.152631.r240234

**Published:** 2024-02-05

**Authors:** Abhilash Thatikala

**Affiliations:** 1 UAMS College of Medicine, Little Rock, Arkansas, USA

I appreciate the author for writing this manuscript. The article presents a case report of a 22-year-old patient with Langerhans cell histiocytosis (LCH) affecting the cervical spine. The patient initially presented with nape pain and radiculopathy, and the diagnosis was confirmed through imaging and biopsy. The patient underwent a successful surgical procedure to remove the tumor in the C3 vertebral body, resulting in complete pain relief and no evidence of new tumor development one month later. The report emphasizes the rarity of LCH in the cervical spine and underscores the significance of prompt diagnosis and treatment for optimal outcomes.

 Below are my comments for the author:

 Abstract : Well written and concise. Please mention the which side the patient had radicular symptoms. 

 Intro:  Well written.

 Case report: Please describe the radicular symptoms as mentioned in the abstract. Please provide histopathology slides if available.

 Discussion: Well written.

 Conclusion: Agree with the comments that this cannot be generalized to broader population. 

 Some potential strengths of the article include:

1. The article presents a unique case report of Langerhans cell histiocytosis involving the cervical spine in an adult patient, which could contribute to the understanding of the disease and its management.
2. The article provides a detailed description of the surgical procedure performed to treat the patient's condition, which could be useful for clinicians and researchers interested in this area. 
  Some limitations of the article may include:
3. The article is based on a single case report, which limits the generalizability of the findings to a broader population.
4. The article may not provide information on the long-term outcomes of the patient, such as recurrence of the disease or complications, which could impact the assessment of the treatment efficacy.

Are enough details provided of any physical examination and diagnostic tests, treatment given and outcomes?

Yes

Is the case presented with sufficient detail to be useful for other practitioners?

Yes

Is sufficient discussion included of the importance of the findings and their relevance to future understanding of disease processes, diagnosis or treatment?

Yes

Is the background of the case’s history and progression described in sufficient detail?

Yes

Reviewer Expertise:

Neurology

I confirm that I have read this submission and believe that I have an appropriate level of expertise to confirm that it is of an acceptable scientific standard.
